# Quality of life for patients with advanced gastrointestinal cancer randomised to early specialised home-based palliative care: the ALLAN trial

**DOI:** 10.1038/s41416-024-02764-x

**Published:** 2024-07-01

**Authors:** Anders Bojesson, Eva Brun, Jakob Eberhard, Mikael Segerlantz

**Affiliations:** 1 Department of Clinical Sciences Lund, Oncology and Pathology, Faculty of Medicine, Lund University, Lund, Sweden; 2 Department of Palliative Medicine, Blekinge Hospital, Karlskrona, Sweden; 3 Department of Oncology, Skåne University Hospital, Malmö, Sweden; 4 Department of Clinical Sciences Lund, Respiratory Medicine, Allergology and Palliative Medicine, Institute for Palliative Care, Faculty of Medicine, Lund University, Lund, Sweden; 5 Department of Palliative Care and Advanced Home Health Care, Primary Health Care Skåne, Region Skåne, Lund, Sweden

**Keywords:** Gastrointestinal cancer, Quality of life

## Abstract

**Background:**

The primary aim of specialised palliative care (SPC) is to improve the quality of life (QoL) for patients with a high symptom burden from a life-threatening disease. This randomised study aimed to assess the QoL impact of early integration of SPC alongside tumour-specific palliative treatment in patients with gastrointestinal (GI) cancers.

**Methods:**

We randomly assigned ambulatory patients with advanced GI cancer to early integration of SPC and palliative tumour-specific treatment or tumour-specific treatment alone. The primary endpoint was QoL assessed at baseline and every sixth week using the Functional Assessment of Cancer Therapy—General (FACT-G) questionnaire.

**Results:**

A total of 118 patients were randomised. The difference in total FACT-G score between patients assigned to early integration with SPC and controls was 5.2 points (95% CI: −0.1 to 10.5, *p* = 0.216), 6.7 points (95% CI: 0.2 to 13.3, *p* = 0.172), and 13 points (95% CI: 5.7 to 20.2, *p* = 0.004) at weeks 6, 12, and 24, respectively.

**Conclusions:**

This prospective randomised trial strengthens the argument for early integration of SPC with tumour-specific treatment in patients with advanced GI cancers. We found an improved QoL for patients with advanced GI cancer 24 weeks after randomisation to early integration of home-based SPC.

**Clinical trial registration:**

ClinicalTrials.gov (ref: NCT02246725).

## Introduction

The primary aim of palliative care (PC) is to improve the quality of life (QoL) for patients with a life-threatening disease; it is mainly advocated for those with advanced cancer [1]. While there is growing support for the early integration of PC alongside tumour-specific treatment in outpatient care of patients with advanced cancer [2] and a growing consensus that early referral (within 8 weeks of diagnosis) improves QoL [3], there is no consensus on the optimum delivery of care or how the expertise of the multiprofessional team is best used.

Varying delivery modes and intensities of PC are currently in use, and the majority of patients are seen at outpatient clinics [4]. A Cochrane review from 2013 states that the evidence regarding the effect of home-based PC on QoL is inconclusive [5]. More research is needed regarding home-based specialised palliative care (SPC), as there is a lack of controlled clinical trials investigating how the resources should be optimally employed [6].

The randomised clinical trial (RCT) by Temel et al. in 2010 is usually referred to as a landmark study, showing gains in both QoL and survival for patients with advanced lung cancer undergoing tumour-specific treatment and randomised to early PC delivered on an outpatient basis, compared to tumour-specific treatment alone [7]. In a later study by the same researchers, patients with newly diagnosed incurable lung or non-colorectal gastrointestinal (GI) cancer were again randomised to tumour-specific treatment with or without early integration of PC. The study confirmed that early integration of PC improved QoL, but the results differed between the two cancer diagnoses. An exploratory subgroup analysis demonstrated continuous improvements in QoL from baseline to 24 weeks after randomisation in patients with lung cancer, whereas in patients with GI cancer no improvement in QoL was found at 12 or 24 weeks after randomisation [8]. The Danish Palliative Care Trial, which investigated a mixed population of patients with advanced cancers randomised to SPC or no SPC alongside tumour-specific treatment, also failed to show any QoL improvement 8 weeks after randomisation [9]. A Cochrane review of studies on early palliative care starting around the time of diagnosis of an incurable cancer found improvements in patients’ symptom control and QoL, as well as a positive impact on anxiety, depression, and overall survival [4].

The present study was performed in Sweden, where basic PC is administered by a family physician through the patient’s healthcare centre, at home, or (and mainly) at an assisted living facility. Access to SPC is provided through referral to these units, predominantly operating in home-based settings and staffed by multidisciplinary specialised teams. We sought to investigate the impact on QoL of early integration of home-based SPC in patients with advanced cancer who were starting palliative chemotherapy.

## Aim

The objective of this randomised study was to assess the impact of early integration of specialised home-based PC alongside tumour-specific treatment by studying the effects on QoL at 6, 12, and 24 weeks following randomisation, and with a last assessment before death, in patients with GI cancers receiving first-line palliative chemotherapy (oesophageal, gastric, hepatobiliary, and pancreatic cancer) or second-line chemotherapy (colorectal cancer).

## Materials and methods

This prospective randomised clinical trial recruited patients between December 18, 2014 and April 29, 2021 at a tertiary cancer centre in the southern Swedish healthcare region. To be included, patients had to live within the catchment area of the two major specialised palliative care units, representing 0.5 million residents. The last date of follow-up was March 1, 2023.

### Study protocol

Patients with upper GI cancer (oesophageal, gastric, hepatobiliary, and pancreatic cancer) eligible for first-line palliative chemotherapy and patients with lower GI cancer (colorectal cancer) eligible for second-line palliative chemotherapy were invited to this nonblinded randomised controlled trial. Patients diagnosed with colorectal cancer were invited to the study when they became eligible for second-line treatment, as we considered that their expected survival rates would then be similar to those of patients with upper GI cancers. After agreeing to participate, patients were randomised 1:1 to either intervention with early integration of home-based SPC alongside tumour-specific treatment, or tumour-specific treatment alone. Patients in the active arm met with the SPC team at home within 6 weeks after randomisation, and at least monthly after that. Patients in the control group were not scheduled to meet with the SPC team unless referred by the treating oncologist; they remained in the control group throughout the analysis, regardless of whether and when they were referred to SPC. All participating patients provided their signed informed consent.

### Inclusion criteria

This study recruited adult (>18 years) ambulatory patients (World Health Organisation performance status [PS]: 0–2) with advanced upper GI cancer confirmed by histological verification. Patients referred for palliative chemotherapy as part of their first-line treatment were invited to participate. Similarly, patients with histologically confirmed advanced lower GI cancers were offered enrolment when they became eligible for second-line palliative chemotherapy. The treating oncologist determined eligibility for participation.

### Exclusion criteria

Patients with ongoing palliative chemotherapy at randomisation (except those with lower GI cancer) were excluded, as were those already included in SPC. In addition, patients with neuroendocrine tumours were excluded, as their treatment options and outcomes differ from those of patients with carcinomas.

### Measures

Quality of life was evaluated with the Functional Assessment of Cancer Therapy—General (FACT-G) questionnaire, measuring health-related QoL in four dimensions according to physical, functional, emotional, and social well-being over the past week [10]. The total score of FACT-G ranges from 0 to 108 points, with a higher score indicating better QoL. Scores were only calculated if at least 80% of the questions were answered, Changes of 4–7 points are considered to comprise a minimally important difference, while changes of 9–14 points indicate a medium-sized clinically significant effect [11]. In addition, mood was assessed with the Hospital Anxiety and Depression Scale (HADS) [12]. The patient-reported HADS questionnaire has two subscales of seven items each, screening for anxiety (HADS-A) and depression (HADS-D), respectively. Subscale scores range from 0, indicating no distress, to 21, indicating maximum distress. A score of 7 or lower on either HADS subscale is considered to be normal, 8–10 points indicates mild distress, and 11–21 points indicates moderate-to-severe distress. Participants completed baseline questionnaires (FACT-G and HADS) at the randomisation occasion, after being randomised. Follow-up assessments were performed every 6 ± 1 weeks until the patient died.

Demographic data were recorded, including age, gender, diagnosis, enrolment date in palliative care, and chemotherapy use. PS at the time of inclusion and the number of contacts, homecare visits, and telephone calls with SPC were extracted from medical records.

### Intervention

Patients in the active study group received planned tumour-directed treatment at the outpatient unit at the Department of Oncology and were also evaluated at their homes by an SPC physician and a palliative care nurse within 6 weeks of randomisation. Services from the rest of the multi-professional team (dieticians, occupational therapists, counsellors, and physiotherapists) were initiated when needed. Patients were offered consultations and homecare around the clock depending on their requirements, and were admitted for inpatient care at the affiliated SPC ward if needed. The SPC team provided advanced homecare services such as administering intravenous fluids including antibiotics, nutritional support, and blood products, assessing symptoms, and monitoring pain management and side effects of medications or oncological treatment. Admittance to inpatient care at the SPC ward could be arranged, generally for a shorter period, for symptom management and to allow respite for caregivers at home. A structured study report was used to assess patients in the active study group every 6 weeks during a home visit according to the study protocol; this included questions regarding prognosis, symptom control, anti-tumoural treatment, patients’ knowledge about their incurable disease, and patients’ ability to live normally and experience satisfaction in everyday life (Appendix 1). A comprehensive systematic symptom assessment using the Integrated Palliative Care Outcome Scale was performed at the same visit [13]. All medical care, including SPC, was financed within the social security system. The control group received the tumour-specific therapy and a referral to the SPC team when deemed appropriate by the treating oncologist.

### Statistics

Assuming a difference of 6 points on the FACT-G scale between the active and control groups, with a standard deviation of 11 in both groups, it was estimated that 108 patients were needed to detect a significant difference with 80% power at a 5% significance level. To compensate for drop-out and mistakenly included patients, a total of 124 patients were included in the trial. Patients’ characteristics at randomisation were described with absolute and relative frequencies for categorical variables, mean with standard deviation for normally distributed continuous variables, and median with range for non-normal variables. Differences between the two study groups in FACT-G and HADS scores at baseline were assessed with Welch’s *t* test. The mean changes in FACT-G, HADS-A, and HADS-D from baseline to weeks 6, 12, 24, and last assessment were computed and compared between the two groups using Welch’s *t* test. The Bonferroni correction was used for each outcome to adjust the *p* values for multiple comparisons. All comparisons were performed on an intention-to-treat analysis. Median overall survival was estimated with the Kaplan–Meier method, with patients alive at the last follow-up (March 1, 2023) being censored at that date, and survival in the two arms was compared with a log-rank test. All statistical analyses were performed with R 4.2.2.

## Results

### Demographics

**Fig. 1:**
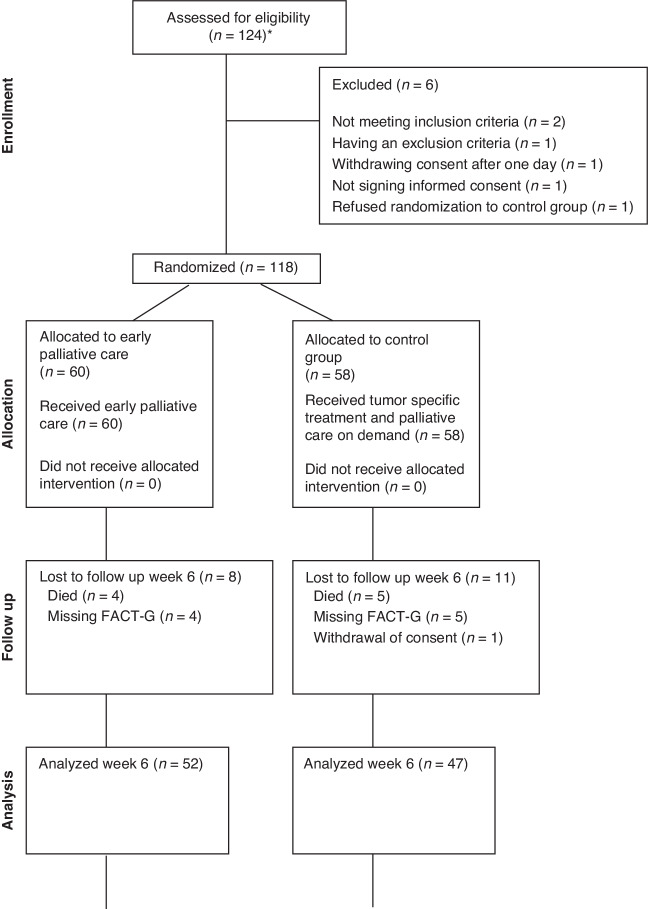

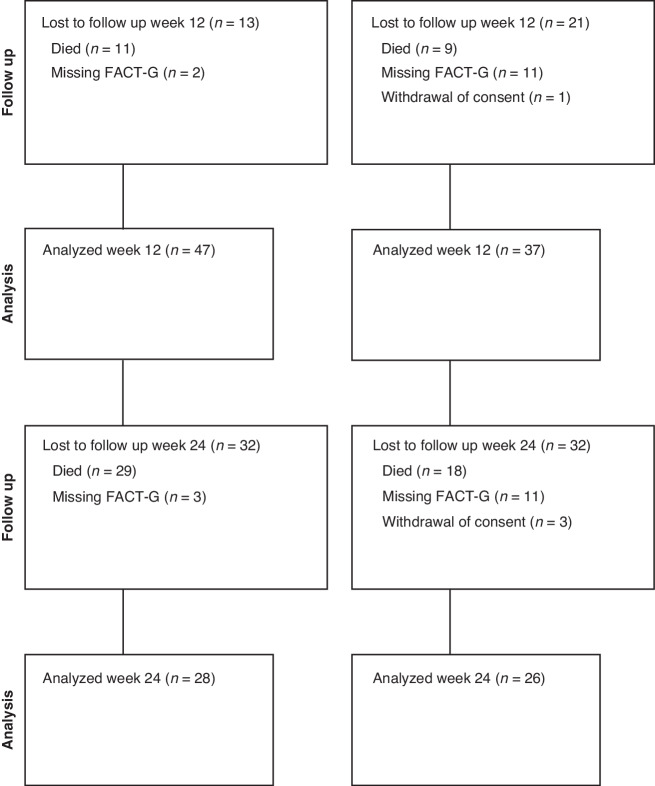
Consort diagram. A flow chart of eligible patients and patients lost to follow-up at weeks 6, 12, and 24. The study did not have an exclusion log. Initial inclusion comprised 124 patients, 6 of whom were then excluded due to not meeting the inclusion criteria (*n* = 2), meeting one of the exclusion criteria (*n* = 1), withdrawing consent one day after randomisation (*n* = 1), refusing randomisation to the control group (*n* = 1), and not signing informed consent (*n* = 1). Six patients withdrew consent during the trial, with one patient doing so after the analysis at week 24.

Initial inclusion comprised 124 patients. Six patients were excluded due to not meeting the inclusion criteria (*n* = 2), meeting one of the exclusion criteria (*n* = 1), withdrawing consent one day after randomisation (*n* = 1), refusing randomisation to the control group (*n* = 1), and not signing informed consent (*n* = 1) (see consort diagram in Fig. 1). Hence, 118 patients were randomised: 55 women with a median age of 70 (45–83) years and 63 men with a median age of 74 (49–85) years. Six patients later withdrew their consent and were therefore only included in the survival analysis. Baseline PS was 0 in 18 (30%) and 26 (45%) patients, 1 in 28 (47%) and 21 (36%) patients, and 2 in 14 (23%) and 11 (19%) patients in the active study group (*n* = 60) and control group (*n* = 58), respectively (Table 1).

**Table 1:** Baseline characteristics of the study participants, specialised palliative care use data, and overall survival.

Variable	Whole study cohort (*n* = 118)	Active group (*n* = 60)	Control group (*n* = 58)
Age in years, median [range]	71.5 [45, 85]	71 [51, 83]	72 [45, 85]
Female gender, *n* (%)	55 (46.6)	24 (40.0)	31 (53.4)
Cancer diagnosis, *n* (%)			
Pancreatic	66 (55.9)	35 (58.3)	31 (53.4)
Hepatobiliary	25 (21.2)	11 (18.3)	14 (24.1)
Gastric	11 (9.3)	7 (11.7)	4 (6.9)
Colorectal	9 (7.6)	4 (6.7)	5 (8.6)
Oesophageal	7 (5.9)	3 (5.0)	4 (6.9)
WHO performance status, *n* (%)^a^			
0	44 (37.3)	18 (30.0)	26 (44.8)
1	49 (41.5)	28 (46.7)	21 (36.2)
2	25 (21.2)	14 (23.3)	11 (19.0)
Palliative chemotherapy lines, *n* (%)^b^	**(*n*** = **112)**	**(*n*** = **60)**	**(*n*** = **52)**
0	2 (1.8)	1 (1.7)	1 (1.9)
1	66 (58.9)	34 (56.7)	32 (61.5)
2	27 (24.1)	14 (23.3)	13 (25.0)
3	17 (15.2)	11 (18.3)	6 (11.5)
Anticancer therapy, *n* (%)^c^	**(*n*** = **110)**	**(*n*** = **59)**	**(*n*** = **51)**
Nab-paclitaxel/Gemcitabine	25 (22.7)	14 (23.7)	11 (21.6)
FOLFIRINOX	24 (21.8)	12 (20.3)	12 (23.5)
Gemcitabine	24 (21.8)	13 (22.0)	11 (21.6)
Combination chemotherapy	14 (12.7)	8 (13.6)	6 (11.8)
FOLFOX	12 (10.9)	8 (13.6)	4 (7.8)
GEMOX	8 (7.3)	2 (3.4)	6 (11.8)
Single-agent chemotherapy	3 (2.7)	2(3.4)	1 (2.0)
Number of chemotherapy cycles, median [range]	6.0 [0.0, 34.0]	6.0 [0.0, 34.0]	6.0 [0.0, 33.0]
	**(*n*** = **118)**	**(*n*** = **60)**	**(*n*** = **58)**
Baseline FACT-G, mean (SD)^d^	71.3 (14.8)	70.2 (14.9)	72.4 (14.9)
Baseline HADS-A, mean (SD)^e^	5.8 (3.8)	5.1 (3.4)	6.5 (4.0)
Baseline HADS-A, *n* (%)			
Normal (0–7)	80 (71.4)	43 (76.8)	37 (66.1)
Mild (8–10)	17 (15.2)	8 (14.3)	9 (16.1)
Moderate/severe (11–21)	15 (13.4)	5 (8.9)	10 (17.9)
Baseline HADS-D, mean (SD)	6.2 (2.8)	6.0 (2.7)	6.4 (2.9)
Baseline HADS-D, *n* (%)			
Normal (0–7)	82 (73.2)	43 (76.8)	39 (69.6)
Mild (8–10)	19 (17.0)	9 (16.1)	10 (17.9)
Moderate/severe (11–21)	11 (9.8)	4 (7.1)	7 (12.5)
Days enrolled in SPC, median [range]		167 [4, 987]	39 [0, 1152]
Assessments by SPC, median [range]			
Physician		10 [1, 34]	2 [0, 24]
Nurse		36 [0, 330]	13 [0, 210]
Telephone		22 [0, 79]	4 [0, 135]
Survival in months, median (95% CI)	7.6 (6.0–10.2)	6.6 (4.9–10.7)	8.7 (6.5–12.2)

*FOLFIRINOX* oxaliplatin, irinotecan, leucovorin, and fluorouracil, *FOLFOX* 5-fluorouracil, oxaliplatin, and leucovorin, *GEMOX* gemcitabine and oxaliplatin, *Nab-paclitaxel* albumin-bound paclitaxel.

^a^WHO performance status of 0 indicates that the patient is asymptomatic, 1 that the patient is symptomatic but fully ambulatory, and 2 that the patient is symptomatic and in bed less than 50% of the day.

^b^Six patients withdrew consent and are excluded from this analysis.

^c^Two patients did not start palliative chemotherapy, and six patients withdrew consent and are excluded from this analysis.

^d^Quality of life (QoL) was evaluated with the Functional Assessment of Cancer Therapy—General (FACT-G) questionnaire, measuring health-related QoL in four dimensions according to physical, functional, emotional, and social well-being over the past week. The total score of FACT-G ranges from 0 to 108 points, with a higher score indicating better QoL.

^e^Mood was assessed with the Hospital Anxiety and Depression Scale (HADS). The patient-reported HADS questionnaire has two subscales of seven items each, screening for anxiety (HADS-A) and depression (HADS-D). Subscale scores range from 0, indicating no distress, to 21, indicating maximum distress. A score of 7 or lower on either HADS subscale is considered to be normal, 8–10 points indicates mild distress, and 11–21 points indicates moderate-to-severe distress.

### Diagnosis

Diagnoses were evenly distributed between the active and control groups: pancreatic cancer, *n* = 35 and *n* = 31, hepatobiliary cancer, *n* = 11 and *n* = 14, gastric cancer, *n* = 7 and *n* = 4, colorectal cancer, *n* = 4 and *n* = 5, and oesophageal cancer, *n* = 3 and *n* = 4, respectively.

### Quality of life

**Fig. 2:**
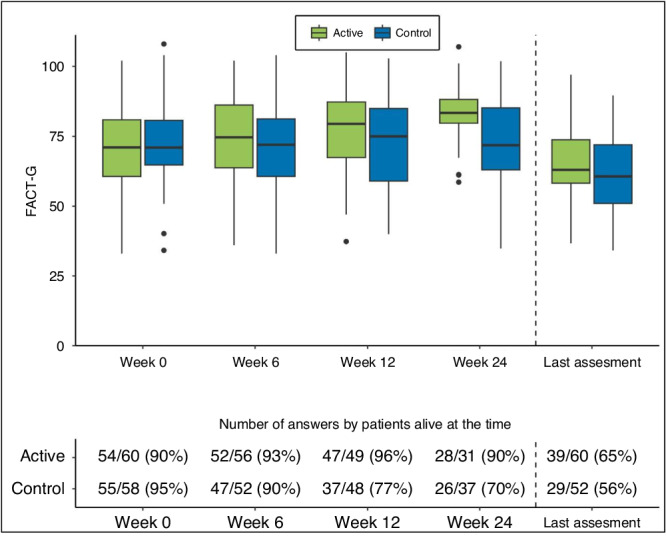
Boxplot showing total FACT-G score in the active and control groups at baseline, weeks 6, 12, and 24 and the last assessment before death. Quality of life (QoL) was evaluated with the Functional Assessment of Cancer Therapy—General (FACT-G) questionnaire, measuring health-related QoL in four dimensions according to physical, functional, emotional, and social well-being over the past week. The total score of FACT-G ranges from 0 to 108 points, with a higher score indicating better QoL The interquartile range (IQR) represents 25–75% of the patients at each measuring point, the whiskers represent 1.5 times the IQR, and the markings outside are outliers. The last assessment before death was made a median of 4.1 weeks (range 0.4–6.7 weeks) before death.

Baseline total scoring of FACT-G at randomisation did not differ between the study groups. At weeks 6, 12, 24, and at last assessment before death, there were 108, 97, 68, and 112 patients alive, respectively, with corresponding response rates on the FACT-G questionnaire of 91.7%, 86.6%, 79.4%, and 60.7% respectively. When looking at the mean total change in FACT-G score compared to baseline, the difference in this change between patients assigned to early integration of SPC and controls was 5.2 points (95% CI: −0.1 to 10.5, *p* = 0.216) at week 6, 6.7 points (95% CI: 0.2 to 13.3, *p* = 0.172) at week 12, and 13 points (95% CI: 5.7 to 20.2, *p* = 0.004) at week 24, with all numerical differences in favour of the early-integration group. At the last assessment, a median of 4.1 weeks (range: 0.4 to 6.7 weeks) before death, the difference between the two groups regarding the mean change in FACT-G score was 3 points (95% CI: −4 to 9.9, *p* = 1.0) (Fig. 2 and Table 2). The mean total FACT-G scores in the active and control groups at weeks 0, 6, 12, and 24 and at the last assessment were 70.2 versus 72.4 points, 74.4 versus 71.5 points, 77.2 versus 73.2 points, 82.6 versus 72.4 points, and 65.9 versus 61.9 points, respectively.

**Table 2:** Changes in FACT-G scores from baseline to weeks 6, 12, and 24 and the last assessment before death.

Outcome	Week	Mean change, active group	Mean change, control group	Difference (95% CI)	*p* Value
FACT-G	6	3.6	−1.5	5.2 (−0.1 to 10.5)	0.216
	12	5.9	−0.9	6.7 (0.2 to 13.3)	0.172
	24	10.8	−2.2	13 (5.7 to 20.2)	0.004
	Last	−6.7	−9.7	3 (−4 to 9.9)	1.000
HADS A	6	−1.3	−0.5	−0.9 (−2.1 to 0.4)	0.728
	12	−0.7	−0.9	0.2 (−1.1 to 1.5)	1.000
	24	−1.4	−1.1	−0.3 (−2.6 to 1.9)	1.000
	Last	0.5	−0.3	0.8 (−1.1 to 2.7)	1.000
HADS D	6	−0.3	0.2	−0.5 (−1.5 to 0.6)	1.000
	12	−0.3	0.3	−0.6 (−1.8 to 0.6)	1.000
	24	−0.8	0.4	−1.2 (−2.6 to 0.3)	0.472
	Last	2.0	0.7	1.4 (−0.4 to 3.2)	0.520

FACT-G scores at weeks 6, 12, and 24 and the last assessment before death were compared to baseline and between the two study groups using a paired *t* test. The Bonferroni correction was used for each outcome to adjust the *p* values for multiple comparisons, and so the values in this table are already multiplied by four. The last assessment before death was made a median of 4.1 weeks (range 0.4–6.7 weeks) before death.

### FACT-G response rates and PS

The response rates for FACT-G at weeks 6, 12, 24, and at the last assessment before death in the active and control groups were 93% versus 90%, 96% versus 77%, 90% versus 70%, and 65% versus 56%, respectively. Response rates were below 80% in the control group at weeks 12 and 24. This led us to investigate PS in responders and non-responders in the control group. At week 12, among the responders (*n* = 37) and non-responders (*n* = 11) in the control group, PS was 0 in 29.7% versus 36.4%, 1 in 43.2% versus 18.2%, 2 in 16.2% versus 18.2%, and 3 in 10.8% versus 27.3%. At week 24, among the responders (*n* = 26) and non-responders (*n* = 11), PS was 0 in 19.2% versus 9.1%, 1 in 42.3% versus 27.3%, 2 in 30.8% versus 27.3%, 3 in 7.7% versus 0%, and 4 in 0% versus 36.4%. These patient groups were small, and no statistical analysis was performed to examine the significance of the differences.

### Hospital Anxiety and Depression Scale

Baseline HADS at randomisation did not differ between the study groups. There were no statistically significant differences in terms of mean change in HADS-A/HADS-D from baseline to any of the measurement points (weeks 6, 12, 24, and the last assessment before death; Tables 1 and 2).

### Specialised palliative care

The median time of enrolment in SPC was 167 (4–987) days in the active group and 39 (0–1152) days in the control group. Fifty (86%) of the 58 patients in the control group were referred to SPC at some point during the disease trajectory. The active and control groups had a median of 10 (1–34) versus 2 (0–24) homecare visits from an SPC team physician, a median of 36 (0–330) versus 13 (0–210) visits from an SPC nurse or other healthcare professional, and a median of 22 (0–79) versus 4 (0–135) telephone calls with any healthcare professional, respectively.

### Chemotherapy

The patients in the active group and the control group received a median of 6.0 (0–34) and 6.0 (0–33) cycles of palliative chemotherapy, respectively (Table 1).

### Overall survival

At analysis, three patients were still alive. The median overall survival was 7.6 months (95% CI: 6.0–10.2) in the total cohort of 118 patients, and 6.6 months (95% CI: 4.9–10.7) in the active study group versus 8.7 months (95% CI: 6.5–12.2) in the control group (*p* = 0.675).

## Discussion

We found an improvement in QoL when integrating home-based palliative care early in the disease trajectory for patients with advanced GI cancers undergoing palliative tumour-specific treatment. The gain in QoL at 24 weeks after randomisation was statistically significant even though the majority of patients in the control group were referred to SPC at some point during the study.

Both the American Association of Clinical Oncology and the European Society for Medical Oncology recommend that diagnosis of an advanced cancer should be followed by early integration of PC alongside standard tumour-specific treatment, in order to manage symptoms and maintain or increase QoL. This recommendation is issued despite the problem of defining how, when, and to whom early integration of PC should be prioritised. Previous studies regarding the early integration of PC have included patients with different diagnoses and exhibited eclectic patterns and intensity of PC. Some patients received around-the-clock home-based SPC, while others received palliative consultations at a clinic or had their consultations by telemedicine, and most studies have reported positive results regarding QoL [2, 7, 9, 14] as a single point measurement.

In two RCTs published in 2010 and 2017, Temel et al. showed that patients with incurable lung cancer gained a significant clinical benefit from early palliative care. In the later study, including patients with advanced lung cancer or non-colorectal GI cancer, a subgroup analysis revealed no positive effect on QoL at weeks 12 and 24 for the GI cancer patient group. In contrast, our results show a significant improvement in QoL, with a difference of 13 points between the early-integration group and controls regarding FACT-G improvement over baseline at 24 weeks. Notably, there are essential differences in our study designs and interventions. In the later study by Temel et al., patients met with the SPC team a mean of 6.5 times, corresponding to once a month over the study period of 24 weeks. Conversely, patients in our active group had a median of 10 visits from a palliative care physician and were in contact with the palliative care team every 2–3 days. To our knowledge, this intense, patient-centred, symptom-based follow-up programme is unique among RCTs dealing with early integration of SPC, and may be the reason for the continuous numerical gains in FACT-G scores seen for patients in the active group. Although this continuous numerical increase in FACT-G scores was not statistically significant at weeks 6 and 12, it indicates that the longer enrolment time with an early integration of SPC is meaningful.

The care environment also differed substantially between our study and the two studies by Temel et al. The latter were both conducted in an outpatient hospital-based SPC environment at a highly specialised institution, whereas our interventions mainly consisted of home visits from the SPC team. Furthermore, patients in our study received all their palliative care from the same PC unit and SPC team from inclusion to death, and were not referred to any other level of care outside the PC department or to a hospice for end-of-life care. An RCT conducted by Eychmüller et al. found no significant improvement in QoL following a single early consultation with an SPC team compared to the standard of care in patients with advanced cancer [15], indicating that one early PC consultation alone is insufficient to improve QoL for patients with advanced cancer.

We found no differences in FACT-G scores between the study groups at the last assessment before death. One reason for this could be that almost all patients in the study cohort were admitted to SPC at some point. Moreover, the time before death for this last assessment differed widely. In retrospect, one can see that our study was not suitably designed to measure QoL at the very end of life.

In line with our results, a recent randomised cross-over study by Kim et al. reported that patients newly diagnosed with metastatic pancreatic cancer (<70 years) had an improved QoL after 16 weeks of SPC compared to baseline [16]. The cohort in that study was similar to the present one, as 91 of our 118 patients (77%) had pancreatic or hepatobiliary cancer. Furthermore, Kim et al. used a multivariate subgroup analysis to show that patients with metastatic pancreatic cancer had a lower QoL score at baseline than those with locally advanced disease, and significant improvement in the total symptom distress score was only found in patients with metastatic cancer. Rodin et al. conducted a secondary analysis of an RCT on early integration of PC in a group of patients with stage IV cancer, finding that independent of cancer diagnosis, improved QoL at 4 months was only seen in patients with a high burden of symptoms at baseline [17].

The results of Kim et al. and Rodin et al. indicate a possibility of selecting patients for early integration of SPC based on symptom severity. Patients with GI cancers, and pancreatic cancer in particular, are regarded to have a high burden of symptoms, and may consequently have more to gain from an SPC team than patients with other malignant diagnoses in the palliative situation, provided a lower burden of symptoms [18, 19]. Thus, an assessment of symptom burden may be one way to select patients for referral to early palliative care. In the present study, baseline FACT-G scores were similar between the two patient groups and were also similar to the scores of cohorts in other studies, and so we consider it unlikely that there was any selection either in the study population or in the randomisation process [8, 20]. However, our cohort displayed lower baseline scores (71.3) than the American reference value for FACT-G in patients with metastasised colorectal cancers (76.9), indicating a higher symptom burden and reduced QoL in our patients [21].

In the present study, we measured QoL every 6 weeks to explore the hypothesis that a longer time of enrolment in SPC would correlate with the improvement in QoL compared to baseline. The repeated assessments weakened the statistical power because of the need to adjust p-values in order to keep a family-wise error rate at ≤0.05 within each outcome. The p-value had to be adjusted to the four analyses, and hence was multiplied by four. Despite this, the study showed a clinical benefit of admitting advanced GI cancer patients to SPC early in the disease trajectory. We believe that the longer time of enrolment allows for a thorough work-up with early detection and treatment of cancer- and treatment-related symptoms, clear communication with caregivers and family, maintenance of QoL, and meaningful end-of-life discussion. The prognosis for these patients is dismal, and healthcare professionals and patients often overestimate the remaining time in life [22]. If time from enrolment to death is short, the care will need to focus on symptom control and end-of-life care. In the control group of the present study, all but eight patients were admitted to SPC, with a median enrolment time of 39 (1–1152) days. As all analyses were based on the intention to treat, we can conclude that this period of time was not enough to abolish the difference in QoL between the two randomisation groups. The gain in mean total FACT-G scores, with a difference of 13 points between the groups at week 24, can be regarded as clinically meaningful, far exceeding the minimal important difference of 4–7 points [11].

Our study has several limitations. We did not establish an exclusion log, and recruitment of the intended number of patients took a long time. It is possible that the treating physicians had different attitudes to the study, and were not always willing to discuss palliative care at their patients’ first visit to the oncology department. The contra-positioning in starting a tumour-specific treatment with the hope of disease control, and at the same time having a discussion about involving the palliative team, could be challenging and time-consuming. As we do not have an exclusion log, we can only speculate on this. These issues, including a temporary study break due to resource allocations during the COVID-19 pandemic, were likely factors in the study’s long inclusion period (6½ years).

In addition, the low number of patients in our study may have led to the absence of statistically significant effects of SPC on QoL after 6 and 12 weeks. The response rate for the FACT-G questionnaire at 12 weeks was low in the control group (77%, 37/48), and the non-responders had a poorer PS at this time point, with PS 3 seen among 27% (3/11) of the non-responders and only 11% (4/37) of the responders. These figures are too small for a statistical analysis, but could indicate that non-responders lacked the energy to respond. There might also have been a selection bias. The distribution of diagnoses in patients with GI cancers receiving palliative chemotherapy was skewed, as the number of patients with colorectal cancers on second-line palliative therapy was unexpectedly low. We believe this is partly because colon cancer patients may have initially received neo-adjuvant chemotherapy, which might have caused confusion among the treating physicians regarding the number of palliative chemotherapy lines given before possible inclusion in the ALLAN study.

Our study has several strengths. This statistically challenging RCT study model provided us the opportunity to study not only the effect of early palliative care but also the process of care, with repeated measurement at multiple time points, and thus the sustainability of the interventions over time. Furthermore, our SPC model allowed us to study the impact of high-quality care with intense interventions in a group of patients with a high burden of symptoms and a short expected survival time.

A 2014 report from the World Health Assembly emphasises that PC tailored to individual needs is an ethical obligation for both governments and healthcare professionals, extending across the continuum of care [23]. Similarly, the International Association of Hospice and Palliative Care has underscored the necessity of universal access to PC, regardless of age, throughout the progression of illness and across all healthcare settings from primary to tertiary care [24]. In line with these recommendations, our model of providing individualised home-based SPC is plausible for both low- and high-income countries.

Differences in the applied methods and endpoints in studies on the early integration of SPC limit the ability to compare results. Our study model, with frequent SPC home visits tailored to patient needs, improved QoL 24 weeks after randomisation. However, this approach is resource-demanding and could entail a limitation on the number of patients admitted to an SPC unit. Thus, there are obvious unsolved issues regarding which components of PC are most important in early integration, and how PC is delivered in order to achieve the best outcomes regarding QoL, symptom control, and cost-effectiveness.

## Conclusion

This prospective randomised trial of home-based PC strengthens the argument for early integration of SPC with tumour-specific treatment in patients with advanced GI cancers. We found an improved QoL for patients with advanced GI cancer 24 weeks after randomisation to early integration of SPC.

### Supplementary information

Appendix 1

## Data Availability

The datasets generated and analysed during the current study are not publicly available due to the data protection law in Sweden. The datasets are available from the corresponding author upon reasonable request.
