# Supplementary material for: Quality of life for patients with advanced gastrointestinal cancer randomised to early specialised home-based palliative care: the ALLAN trial
Source: Br J Cancer. 2024 Jul 1;131(4):729–36. doi: 10.1038/s41416-024-02764-x (PMC11333621; doi:10.1038/s41416-024-02764-x)

Appendix 1

Structured visit report for patients randomized to early integration of specialized palliative care – the ALLAN trial

Weeks after randomization: Personal identification number:

Date (YYMMDD): Initials:

Reporting healthcare professional:

A structural assessment of symptoms and other needs to be performed at the first visit and at least every six weeks thereafter for patients randomized to early integration of specialized palliative care (SPC). Every question listed below is answered with **Yes** or **No**, apart from question 3, which can also be answered with **Not relevant** if the patient has no ongoing tumor-specific treatment (steroids are not considered a tumor-specific treatment). Questions in *italics* are meant as support, and are examples of questions to ask to find the proper answers to the main questions (1–5). Helping the patient is more important than focusing on these five questions. Comments can be noted in the commentary field.

**Frequency of follow up**

- Every 6 ± 1 weeks
- SPC team visit with at least a physician (specialized in palliative care) and a nurse.

**The following symptom assessment tools should be filled out at each structural assessment:**

- Integrated Palliative care Outcome Scale (IPOS). If the patient is unable to fill out the IPOS, a healthcare professional should instead fill out the Edmonton Symptom Assessment System – revised (ESAS-r)
- Palliative Performance Scale (PPSv2). The PPSv2 is included at the end of this document, and the result (%) should be marked as the answer to question 6.

**Structured assessment of symptoms:**

**1. Does the patient understand the meaning of their disease and prognosis? Yes No**

- *Does the patient have questions about their disease?*
- *Has the patient understood that it is not possible to cure their disease?*
- *Does the patient have reasonable expectations about their prognosis?*
- *Has the patient understood the meaning of palliative treatment?*

**Comments from the SPC team: ______________________________**

**2. Does the patient have symptom control? Yes No**

- *Check the results from the filled-in IPOS.*
- *Does the patient have questions about their symptoms?*
- *Go through prescribed medications together with the patient. Are the medications reasonable?*

**Comments from the SPC team: ______________________________**

**3. Does the patient have an effect or benefit from ongoing cancer-specific therapy?**

**Yes No Not relevant**

- *What thoughts does the patient have about their ongoing tumor-specific treatment?*
- *What are the side effects in relation to anticipated gain? Does the patient have any questions about this?*

**Comments from the SPC team: ______________________________**

**4. Does the patient have the possibility to live life and experience activities that bring joy? Yes No**

- *Does the patient need psychological or spiritual counseling?*
- *How do the caregivers handle the patient’s disease (according to the patient)?*
- *Is there anyone in the family in need of psychological or spiritual counseling (according to the patient)?*

**Comments from the SPC team: ______________________________**

**5. Has future care within SPC been discussed with the patient? Are there any planned meetings with healthcare professionals outside the SPC team? Yes No**

- *What is the plan for future care for the patient within SPC? Symptom control? Ongoing medications? Do other SPC team members (dieticians, occupational therapists, counselors, and physiotherapists) need to get involved?*
- *Does the patient have planned appointments with the Department of Oncology? Is there any planned computed tomography or other imaging? Are there any other medical procedures planned?*

**Comments from the SPC team: ______________________________**

**6. PPS score ______%**


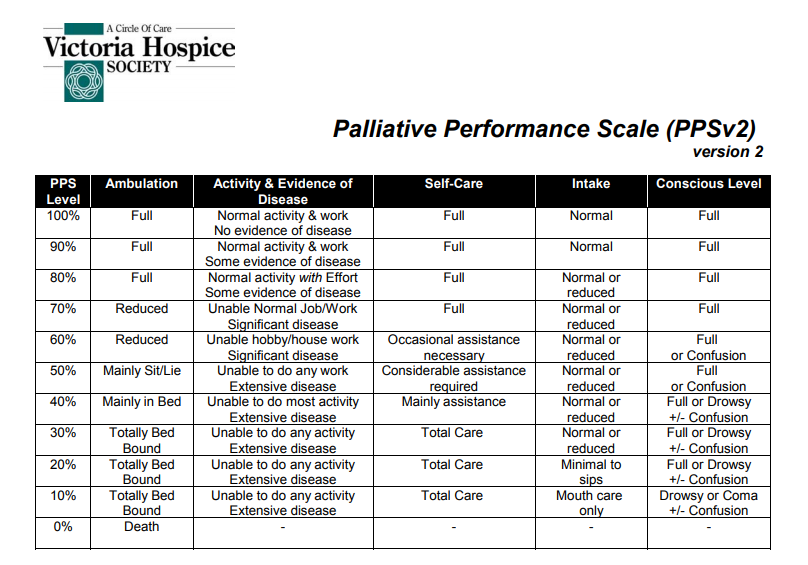

Supplement: Supplementary file 1 — Appendix 1 [file 41416_2024_2764_MOESM1_ESM.docx]
